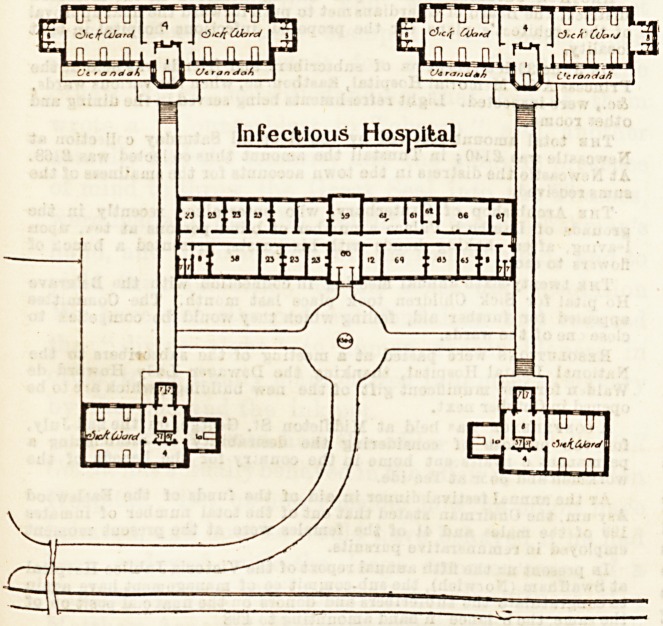# Royal Victoria Hospital, Montreal

**Published:** 1893-07-29

**Authors:** 


### The Institutional Workshop.

### HOSPITAL CONSTRUCTION.

### ROYAL VICTORIA HOSPITAL, MONTREAL.

The hospital whose plan we illustrate to-day owes its existence to the generosity of Lord Mountstephen and Sir Donald Smith, K.C.M·G., two distinguished Canadians, whose names are so widely known in connection with the Canadian Pacific Railway.

It was originally intended that the hospital should be built on a site given for the purpose by the Corporation of Montreal, but objection being taken by the inhabitants to the ground being used for a hospital, the present site to the north-east of the original one was bought. It would appear that though the site is somewhat narrow the land originally given to the hospital still remains the property of the institution, though it can only be used as a recreation ground. It thus secures for the benefit of the patients a large open space to the south and west.

The narrowness of the site, however, presented far less difficulty to the architect than its contour, the ground rising no less than 180 feet in the length, and from 30 to 80 feet in the breadth of the site. This difficulty has been got over by making the main entrance for patients midway up the slope on which the main building stands, thus permitting one floor of the various blocks to be reached on the same level.

The main building will, when completed, comprise thirteen distinct blocks connected by bridges. The ward blocks are arranged along the north-east and south-west boundaries. Between these is the administrative block, in which are also the out-patients' department and receiving-rooms. The entrance to this last building is approached by carriage and footways from Pine Avenue, through a covered gateway and porter's lodge. The centre portion contains on the ground floor the board-room, the secretary's office, the medical officers' sitting and bed rooms, and kitchen, &c, and the porter's-room and principal staircase. The upper floors contain a large number of bed-rooms for the nurses—a separate room for each—together with their common sitting and dining rooms, library, linen and bath rooms, lavatories, &c.

The Lady Superintendent's apartments are placed on the first floor. The general kitchen for the whole of the buildings is placed on the topmost floor of the centre of this building, and the scullery and accessory stores, larders, pantries, &c. The wardmaids and servants' rooms are in the adjoining roof floors. A service staircase and coal and food lifts communicate with all floors, so that the meals can be distributed to the wards in each and every block without difficulty or undue delay, or the necessity of ascending or descending staircase.

The rear extension of this block contains on the basement floor boiler-room, coal and other stores, and on the ground floor the dispensary and clinical lecture-room, and also the medical officers' and Lady Superintendent's offices. On the upper floors are additional nurses' bed-rooms. The two large projecting blocks on the south-west and north-east boundaries each contain three large wards for the accommodation of 32 patients in each. The north-east block is devoted to medical, and the south-west block to surgical patients. Attached to each large ward are the nurses and medical officers' rooms, the ward kitchen, and a separation ward for two beds. The bath-rooms and ward offices are contained in the round towers at the ends of the wards, which, it will be noted, are so placed as not to interfere appreciably with the outlook from the large end windows and the balconies provided for the use of the patients.

Adjoining each ward block, and connected with them by cross ventilated bridges (which in summer time would be quite open), is the staircase block, containing a broad and easy-going staircase, patients' lift, patients' clothing and linen stores, &c. In the centre is the large ventilating shaft which draws the foul air from all the adjoining wards. Up the centre of this shaft is carried the smoke shaft from the boilers in the basement, which materially assists the draught power.

Beyond the staircase block on the south-west side, and connected with it as before by bridges, are the women's and children's wards. The latter are on the first floor level, and comprise a large ward for twelve beds, a separation ward for two beds, and ward kitchen, nurses and medical officers rooms, &c.

The wards devoted to women's diseases are contained on the second and third floors, and extend over the top floors of the staircase block and a small part of the adjoining ward block. They comprise a large gynæcological ward for twelve beds, lock ward for six beds, and separation ward for two beds, four wards for two, and one for one abdominal case, also ward kitchens, nurses' rooms, &c.

A small operation room and an anæsthetic room are also provided.

It should be noted that all the nurses and medical officers' rooms throughout the blocks are so arranged and finished that they can be used as separation wards if necessary.

The last block on this side is the surgical theatre, which will seat 250 students. The professor's private rooms, students' hall and instrument rooms are immediately contiguous on the ground floor of the last described block. Here also are an anæsthetic and an after-recovery room, and attendants' room between them.

Returning to the buildings on the north-east boundary (medical side) on the first floor of level of the staircase block is a children's ward for six beds, and on the top floor are male and female erysipelas wards, comprising four male and four female wards each for one bed, with ward kitchen and nurses' room adjoining.

The next block is the medical theatre for 200 students; the adjoining professor's private room, clinical chemistry room, and patients' waiting room being on the ground floor of the staircase block.

Beyond the theatre is another block of two additional wards for ophthalmic cases, each containing sixteen beds, two rooms containing two beds each for medical cases, one ward divided into compartments for thirteen opthalmic cases, and two separation wards, one for two and one for one patient; nurses rooms, ward kitchens, &c., are provided on each floor; as before.

Beyond this building and connected to it as before by bridges is the staircase block similarly arranged to those already described.

Beyond the staircase is a block devoted entirely to paying patients. There are two floors, each divided into twelve separate wards, each for one patient; and on each floor are a nurses' room, a dining or sitting room, and a small kitchen and offices as before.

In the rear of the back extension of the administrative buildings is a detached pathological block, containing on the ground floor the mortuary, mourners' waiting room, staircase, and shell store; and also an icehouse macerating room, cold mortuary, and lift in direct communication with the pathological theatre above.

It will be noted that the roadway for funerals has been so placed as not to pass unneccessarily by any of the ward blocks.

On the upper floor is the theatre capable of seating 100 students, with professor's private room, and work and model rooms.

### ROYAL VICTORIA HOSPITAL, MOUNT ROYAL, MONTREAL.

PLAN OF PRINCIPAL FLOOR.

1. Separation Ward.

1a. Private Patients.

2. Nurse or Ward.

3. Medical Officer or Ward.

4. Ward Kitchen.

5. Bridge.

6. Staircase.

7. W.C.'s, Lavatories, Sinks, &c.

8. Bath Room.

9. Ventilating Shaft.

10. Patients' Own Clothing Store.

11. Fire Escape Staircase.

12. Linen Store.

13. Students' Room.

14. Instruments, &c.

15. Professors' Room.

16. After-Recovery Room.

17. Attendant.

18. Anæsthetic Room.

19. Surgical Theatre (lower part).

20. Students' Hats and Cloaks.

21. Officers' Mess Room.

22. Mess Room Scullery.

23. Nurses' Room.

24. Work Room.

25. Superintendent Night Nurse's Night Room.

26. Service Stairs.

27. Service Room.

28. Lifts.

29. Housemaids' Closet.

30. Waiting Room for Nurses.

31. Dispensary and Drug Store.

32. Lady Superintendent's Office.

33. Medical Superintendent's Office.

34. Medical Admission Room.

35. Entrance Hall and Waiting Room for Out-Patients.

36. Dressing Rooms.

37. Casualty and Operating Room.

38. Consulting Room.

39. Eye Admission Room.

40. Dark Room.

41. Surgical Admission Room.

42. Yard.

43. Shell Store.

44. Waiting Room.

45. Mortuary.

46. Inner Mortuary.

47. Cold Mortuary.

48. Ice House.

49. Macerating Room.

50. Corridor through roof.

51. Female Eye Wards.

52. Male Eye Wards.

63. Female Eye Separation Wards.

54. Paying Patients' Wards.

55. Paying Patients' Dining and Sitting Room.

56. Paying Patients' Kitchen.

57. Cupboard.

58. Nurses' Sitting Room.

59. Medical Officers' and Business Room.

60. Entrance Hall.

61. Stores.

62. Larder.

63. Servants' Hall.

64. Servants' Dormitory.

65. Cook's Room.

66. Kitchen.

67. Scullery.

68. Porter and Footway Entrance.

NOTE.—Figures in circles show finished levels of the roads (Datum line 200 feet above Corporation Datum).

In the rear of the main hospital blocks and at a considerable distance from them is the site of the infectious hospital on the Hut system. It is proposed that this shall consist of five distinct buildings unconnected except by path and roadways. There will be two blocks, each containing two wards for six beds each, and two smaller buildings each containing one ward for four beds, and one isolation ward for one bed. Each block is to have its own ward kitchen, bath-room, &c. The large central block will be devoted entirely to administrative purposes, and comprises an entrance and waiting hall, medical officers' and business room, nurses and servants' bed-rooms, and sitting-rooms, kitchen, servants' hall, bathrooms, &c.

The drainage has been designed upon a carefully arranged system, with glazed earthenware pipes jointed in cement and laid on beds of cement concrete in trenches excavated in the solid rock. Manholes and inspection pits, &c., have been liberally provided in accordance with the latest modern requirements.

The plans were prepared by Mr. H. Saxon Snell, F.R.I.B.A., of London, and the erection of the buildings superintended by Mr. J. R. Rhind, of Montreal.

There are in this, on the whole, carefully thought out plan one or two points which call for criticism. The adoption of the system of coupled beds in the large wards is a mistake which ought not to have been made in so important a building, and one, too, for which funds appear to have been forthcoming without stint. It suggests either ignorance of the principles in which a ward should be designed or an unfortunate restriction in floor space per bed. The planning of the sanitary wings with their useless and wasteful apsidal ends seems to be mere striving after external effect at the cost of internal convenience. The arrangement of the operation theatre and its adjuncts is also very faulty. The anæsthetic room should be in direct communication with the theatre, and the entrance for students entirely separate from that for patients and staff. The circuitous course that a patient under chloroform or ether would have to travel from the anæsthetic room to the theatre will prove most inconvenient in practice, and will probably lead to the abandonment of the practice of anæsthetising patients outside the theatre.

The infectious hospital is not yet built, but it would be advisable to make some radical alterations in the entrances to the various blocks before the work is carried out. As at present planned they are far too confined for practical use.

## Figures and Tables

**Figure f1:**
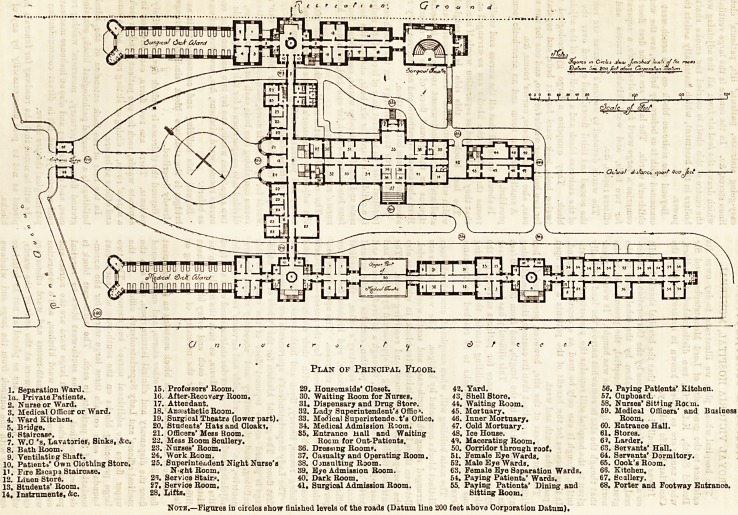


**Figure f2:**